# Stimulation of Host Immune Defenses by a Small Molecule Protects *C. elegans* from Bacterial Infection

**DOI:** 10.1371/journal.pgen.1002733

**Published:** 2012-06-14

**Authors:** Read Pukkila-Worley, Rhonda Feinbaum, Natalia V. Kirienko, Jonah Larkins-Ford, Annie L. Conery, Frederick M. Ausubel

**Affiliations:** 1 Division of Infectious Diseases, Massachusetts General Hospital, Harvard Medical School, Boston, Massachusetts, United States of America; 2 Department of Molecular Biology, Massachusetts General Hospital, Harvard Medical School, Boston, Massachusetts, United States of America; 3 Department of Genetics, Harvard Medical School, Boston, Massachusetts, United States of America; University of California San Francisco, United States of America

## Abstract

The nematode *Caenorhabditis elegans* offers currently untapped potential for carrying out high-throughput, live-animal screens of low molecular weight compound libraries to identify molecules that target a variety of cellular processes. We previously used a bacterial infection assay in *C. elegans* to identify 119 compounds that affect host-microbe interactions among 37,214 tested. Here we show that one of these small molecules, RPW-24, protects *C. elegans* from bacterial infection by stimulating the host immune response of the nematode. Using transcriptome profiling, epistasis pathway analyses with *C. elegans* mutants, and an RNAi screen, we show that RPW-24 promotes resistance to *Pseudomonas aeruginosa* infection by inducing the transcription of a remarkably small number of *C. elegans* genes (∼1.3% of all genes) in a manner that partially depends on the evolutionarily-conserved p38 MAP kinase pathway and the transcription factor ATF-7. These data show that the immunostimulatory activity of RPW-24 is required for its efficacy and define a novel *C. elegans*–based strategy to identify compounds with activity against antibiotic-resistant bacterial pathogens.

## Author Summary

Infections with antibiotic-resistant bacterial pathogens are increasing at an alarming rate, and there are very few new therapies currently being developed. We have identified a small molecule that protects the nematode *C. elegans* from bacterial infection by stimulating the host immune response, not by directly interfering with bacterial growth, which is the mechanism employed by all currently available antibiotics. In this study, we investigate the mode of action of this small molecule and demonstrate that it stimulates the expression of immune effector molecules via evolutionarily conserved immune regulators. These studies illustrate the potential of targeting host immunity for the treatment of bacterial infections and the advantages that *C. elegans* offers both to identify small molecules that target key cellular processes and to study their mechanism of action.

## Introduction

Studies in the model nematode *Caenorhabditis elegans* have greatly expanded our understanding of development, neurobiology, host-pathogen interactions, and many other aspects of metazoan biology. Here we show that *C. elegans*-based assays enable the identification of immunostimulatory compounds, which can be employed together with genetic analyses to interrogate innate immune signaling pathways.

Previously, we demonstrated that *C. elegans* can be used in bacterial infection assays to identify novel antimicrobials [1]–[3]. Fifteen to twenty *C. elegans* animals fit comfortably in the wells of standard 384-well assay plates and the assay can be automated using image analysis software, which enables such studies to be conducted in high-throughput [1]. Using this system, we tested 37,214 compounds and identified 119 small molecules that prolonged the lifespan of nematodes infected with the Gram-positive human bacterial pathogen *Enterococcus faecalis* [1].

We hypothesized that the *C. elegans*-based screen for novel anti-infectives would identify small molecules that cure nematodes by stimulating the host innate immune response, in addition to compounds that block microbial virulence or directly inhibit bacterial growth [1]. In nature, nematodes consume bacteria for food and have evolved sophisticated innate immune mechanisms within their intestinal epithelium to defend against ingested pathogens [4]. *C. elegans* mount specific immune responses toward both bacterial and fungal pathogens using immune signaling mediators that are strongly conserved throughout evolution [5]–[10]. Principal among these regulators is the NSY-1/SEK-1/PMK-1 Mitogen Activated Protein (MAP) kinase pathway, orthologous to the ASK1 (MAP kinase kinase kinase)/MKK3/6 (MAP kinase kinase)/p38 (MAP kinase) pathway in mammals [10]. In *C. elegans*, the p38 MAP kinase pathway acts cell autonomously in the intestine [11] to coordinate the expression of immune effectors such as C-type lectins and genes that may encode antimicrobial peptides [8]. Recently, Shivers et al. found that the transcription factor ATF-7, an ortholog of mammalian ATF2/ATF7, is phosphorylated by the p38 MAP kinase PMK-1 and is also required for defense against ingested bacterial pathogens [12].

In this study, we describe a small molecule named RPW-24 that strongly stimulates the innate immune response of *C. elegans* in a manner that confers a survival advantage for nematodes during bacterial infection. We show that the activity of this compound is partially dependent on the *C. elegans* p38 MAP kinase cassette and ATF-7. These data demonstrate that *C. elegans* can be used in facile *in vivo* screens to identify compounds with desirable biological activities.

## Results

### A Compound with Curing Activity in a Live Animal Infection Model Is Not a Traditional Antibiotic

In a previous study, we screened 37,214 small molecules for those that prolonged the lifespan of *C. elegans* infected with the Gram-positive bacterial pathogen *E. faecalis* as a means to identify novel antimicrobials [1]. Of 119 compounds identified, 31 did not have any structural relationship to known antimicrobials and ten of these small molecules were effective against *E. faecalis* in the *C. elegans* infection model at doses that did not inhibit growth of the pathogen in an *in vitro* growth assay [1]. In contrast, all currently available antibiotics interfere with some aspect of bacterial growth or metabolism. In the *C. elegans*-based assay, traditional antibiotics, such as tetracycline, ciprofloxacin, ampicillin, and vancomycin, cured *E. faecalis*-infected nematodes only at doses several fold higher than the *in vitro* minimum inhibitory concentration (MIC) for bacterial growth [2]. We therefore hypothesized that a subset of these 31 small molecules conferred a survival advantage to infected worms by either stimulating the host immune response of *C. elegans* or by interfering with virulence factor production in the bacteria.

**Figure 1:**
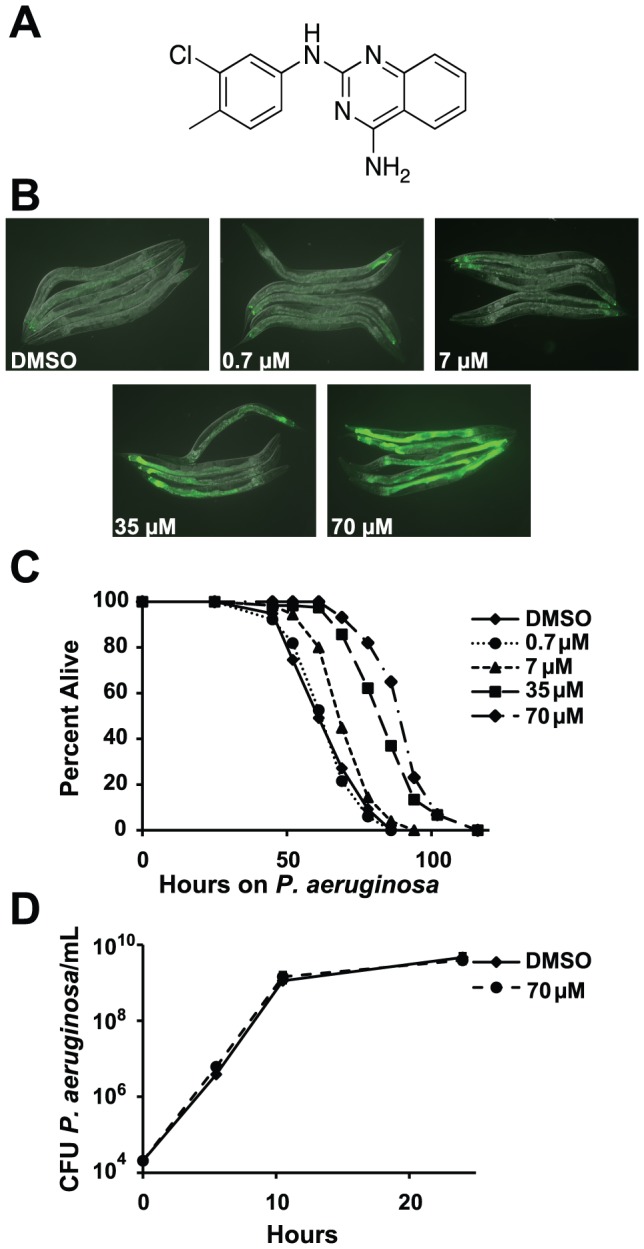
RPW-24 activates *F35E12.5::GFP* and prolongs the lifespan of *C. elegans* infected with *P. aeruginosa* without affect growth of the pathogen. (A) The chemical structure of 2-N-(3-chloro-4-methylphenyl) quinazoline-2,4-diamine, which we have named RPW-24. (B) Fluorescence microscopy images of GFP expression from *C. elegans acIs101* animals, which express a *F35E12.5::GFP* transgene, exposed to the indicated concentration of RPW-24 or DMSO, the solvent control, for 16 hours at 15°C. Green is GFP expression. (C) *P. aeruginosa* infection assay of wild-type nematodes exposed to different concentrations of RPW-24 compared to DMSO. The lifespan extension conferred by 70, 35 and 7 µM RPW-24 compared to DMSO treatment is significant in two biological replicates (*P*<0.0001). Data at each time point are the average of three plates per strain, each with approximately 50 animals per plate (sample sizes are given in Table S2). Data are representative of two independent experiments. (D) RPW-24 does not affect the growth of *P. aeruginosa* in the same media used for the *C. elegans* infection assay (slow-kill media). Data are the average of two biological replicates with error bars representing standard deviation.

We reasoned that a small molecule, which demonstrated curing activity against diverse bacteria without directly affecting bacterial growth, would be a candidate immunostimulator. It would be less likely for such a compound to be a specific inhibitor of bacterial virulence determinants given the complex and presumably pathogen-specific nature of these factors. We therefore screened the 31 compounds described above that did not have any structural relationship to known antimicrobials for those that prolonged the lifespan of nematodes infected with *Pseudomonas aeruginosa*, a Gram-negative pathogen, at doses lower than the *in vitro* MIC for *P. aeruginosa*. We found that eight of the 31 small molecules demonstrated *in vivo* efficacy against nematodes infected with *P. aeruginosa* (Table 1, Figure 1A and Figure S1) at doses several fold lower than the *in vitro* MIC for these compounds against *P. aeruginosa*. Indeed, four of the tested compounds did not affect the growth of *P. aeruginosa* at any concentration tested (Table 1).

**Table 1:** Compounds effective against *P. aeruginosa* in the *C. elegans* assay at doses that do not inhibit bacterial growth.

Compound Number	Effective concentration (µM)	*in vitro* MIC (µM)	*F35E12.5::GFP*
1	3.3	32	−
3	3.3	>264	−
14	5.1	204	−
16	4.8	>192	−
17	8.1	>325	−
18	1.7	33	−
24	11	>351	++
31	1.6	266	−

RPW-24 induced *F35E12.5::GFP* expression and was effective against *C. elegans* infected with *P. aeruginosa* without inhibiting growth of the bacteria. See Figure 1A and Figure S1 for the structures of these compounds. ++ represents strong activation of *F35E12.5::GFP*. MIC equals minimum inhibitory concentration.

To ask if any of these eight compounds could activate the transcription of a putative immune effector, we used transgenic *C. elegans* animals carrying a transcriptional GFP reporter for the gene *F35E12.5* [13]. *F35E12.5* encodes a protein that contains a CUB-like domain and is involved in the transcriptional response towards several bacterial pathogens, including *P. aeruginosa* [8], [13], [14]. Interestingly, one of the four compounds that exhibited *P. aeruginosa* curing activity in the *C. elegans* infection assay without affecting growth of the pathogen (RPW-24, Figure 1A) activated the *F35E12.5::GFP* reporter in a dose dependent manner (Figure 1B). We therefore decided to focus on RPW-24 and explore its mode of action in more detail.

We found that RPW-24 promoted survival of *P. aeruginosa*-infected animals in a dose-dependent manner using an agar-based assay, the typical way that *C. elegans* infection assays are carried out in most laboratories (Figure 1C). *C. elegans* treated with 7, 35 and 70 µM of RPW-24 (but not 0.7 µM) were significantly resistant to *P. aeruginosa* infection compared to animals treated with the solvent control (DMSO)(Figure 1C). To determine if RPW-24 directly affects the growth of *P. aeruginosa*, we monitored *in vitro* bacterial growth in the presence 70 µM RPW-24 and DMSO. We conducted these experiments in the same media used for the nematode killing assay (Figure 1D) or in standard bacterial culture media (Luria broth, data not shown), but in the absence of *C. elegans*. In both cases, we found that 70 µM RPW-24 did not affect the growth rate of *P. aeruginosa* (Figure 1D and data not shown). In the *C. elegans- P. aeruginosa* pathogenicity assay, we observed that the intestines of the DMSO-treated animals were markedly distended and packed with *P. aeruginosa* cells 40 hours after infection (Figure S2), consistent with previous observations [9], [15]. In contrast, animals treated with RPW-24 had non-distended intestines, a morphology that was strikingly different from the DMSO controls (Figure S2).

In summary, RPW-24 is efficacious against both Gram-positive [1] and Gram-negative bacteria (Figure 1C) at concentrations that do not inhibit bacterial growth (Figure 1D, Table 1) and activates the transcription of a putative immune effector, *F35E12.5* (Figure 1B). RPW-24 treatment also dramatically reduced the intestinal burden of *P. aeruginosa* 40 hours after infection compared to DMSO controls (Figure S2). These data suggest that RPW-24 affects the host immune response of *C. elegans* in a manner that confers a survival advantage against infection with diverse bacterial pathogens.

### Exposure to RPW-24 Induces Immune Response Genes in *C. elegans*

**Figure 2:**
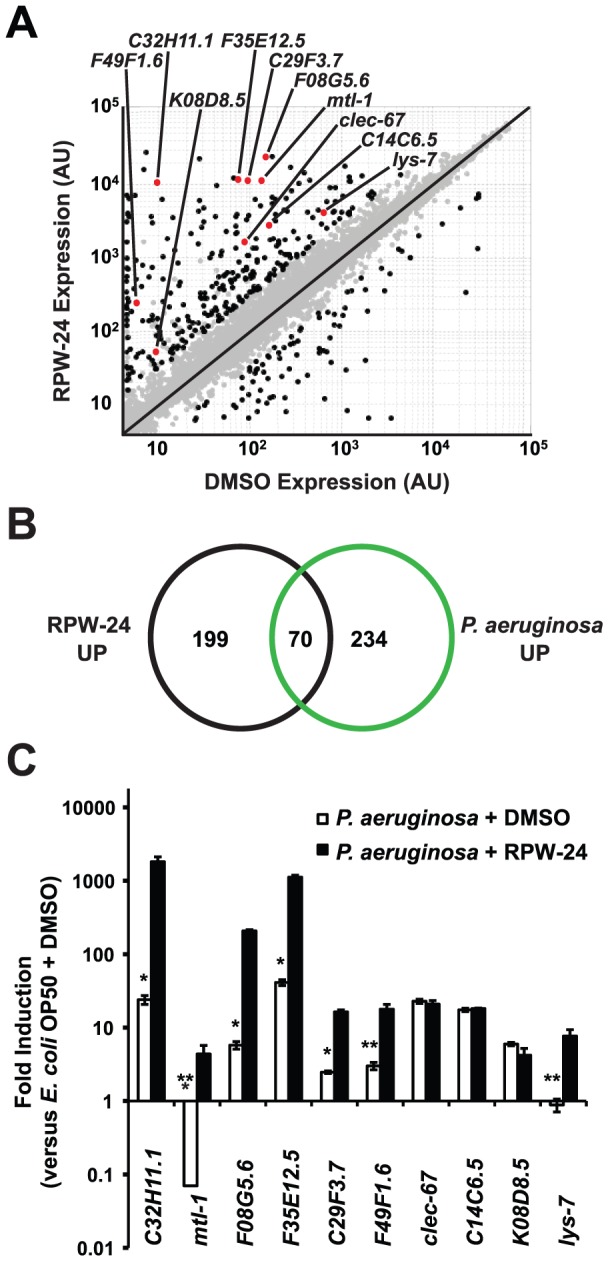
RPW-24 induces the transcription of putative immune effectors in *C. elegans*. (A) A scatter plot compares gene expression levels of all 22,250 sequences on the Affymetrix GeneChip in wild-type *C. elegans* exposed to RPW-24 versus DMSO. Black dots highlight the 269 genes that were induced and the 62 that were repressed at least three-fold (*P*<0.025). Gray dots are genes whose expression levels did not significantly change in this study. The location on the scatter plot of the ten putative immune effectors whose expression was studied further by qRT-PCR are indicated and highlighted with red dots. Genes that fall on the black line are expressed at equal levels in both conditions. AU = arbitrary units. (B) Venn diagram gives the overlap of the *C. elegans* genes induced three-fold or greater by RPW-24 (*P*<0.025) in the microarray analysis (this study) with the genes upregulated during *P. aeruginosa* infection (greater than 2-fold, *P*<0.01) [8]. *P*<2.7×10^−16^ for the degree of overlap between these datasets versus the amount expected by chance alone. (C) Shown are qRT-PCR data of ten putative *C. elegans* immune effectors in wild-type animals infected with *P. aeruginosa* for eight hours and exposed to either 70 µM RPW-24 or DMSO, each plotted versus expression of the indicated genes in *C. elegans* exposed to *E. coli* OP50 and DMSO. The data are the average of two biological replicates each normalized to a control gene with error bars representing SEM. *P<0.05, **P = 0.06 and ***P = 0.08 for the comparison of fold change of the indicated gene in *P. aeruginosa*-infected animals exposed to RPW-24 versus DMSO.

To further study the effects of RPW-24 on *C. elegans*, we used Affymetrix whole genome GeneChips to generate transcriptome profiles of wild-type nematodes following exposure to either 70 µM RPW-24 or the solvent control DMSO in liquid culture media in the absence of bacterial pathogens for 16 hours at 15°C (Figure 2A). We found that RPW-24 induced a remarkably robust transcriptional response that involved only a small fraction (∼1.3%) of the genes of the *C. elegans* genome (Figure 2A). 269 genes were upregulated three-fold or greater (*P*<0.025), 125 of which were induced more than 50-fold during RPW-24 exposure (Table S1). The most highly upregulated gene was expressed more than 3500-fold higher in compound-exposed worms. For confirmation, we used qRT-PCR to analyze 10 genes that exhibited varying degrees of induction and expression levels in the microarray analysis (Figure 2A). We found that the transcriptional changes observed in the transcriptome profiling analysis directly correlated with the values obtained by qRT-PCR for all 10 genes tested (Figure S3). We also observed no difference in *C. elegans* gene induction whether the RPW-24 exposure occurred in liquid or on solid media (Figure S3). Taken together, these data suggest that RPW-24 strongly induces gene transcription in the absence of pathogen exposure.

Examination of the 269 genes that were induced greater than three-fold revealed that RPW-24 causes induction of many genes previously shown to be involved in the *C. elegans* transcriptional response to pathogenic bacteria. We found that 70 of the 269 genes induced greater than 3-fold by RPW-24 were also activated during infection with *P. aeruginosa* (a gene set characterized by Troemel et al. [8])(Figure 2B), which is significantly more than the 3.3 gene overlap expected by chance alone (*P*<2.7×10^−16^). Among these induced genes are several gene classes that have been implicated in antimicrobial defenses, such as CUB-like domain-containing genes, ShK-like toxins, C-type lectins and small molecule kinases (Table S1) [8]. This result is particularly interesting considering that RPW-24 is active against *P. aeruginosa* in the worm infection model but does not affect growth of the pathogen *in vitro*.

The transcriptome profiling analysis demonstrated that RPW-24 induces the transcription of putative immune effectors while *C. elegans* animals are feeding on *Escherichia coli* OP50, a relatively non-pathogenic food source for nematodes. We wondered if RPW-24 would further enhance the induction of these genes when *C. elegans* is infected with a bacterial pathogen that activates expression of these effectors. We therefore used qRT-PCR to test the level of induction of ten putative immune effectors (Figure 2A) during *P. aeruginosa* infection in the presence and absence of RPW-24. This panel included five genes that contain a CUB-like domain (*C32H11.1, F35E12.5, F08G5.6, C29F3.7*, and *K08D8.5*), two ShK-like toxins (*F49F1.6* and *C14C6.5*), one antibacterial lysozyme (*lys-7*), one C-type lectin (*clec-67*) and one metallothionein (*mtl-1*). Eight of these ten genes were activated by *P. aeruginosa* in the absence of RPW-24 (Figure 2C). Interestingly, the induction levels of five of these eight genes (*C32H11.1, F08G5.6, F35E12.5, C29F3.7*, and *F49F1.6*) were markedly increased in the presence of both RPW-24 and *P. aeruginosa* (Figure 2C). RPW-24 also caused the induction of one gene that was strongly repressed during *P. aeruginosa* infection (*mtl-1*) and another whose transcription was unaffected by *P. aeruginosa* exposure (*lys-7*). RPW-24 did not change the level of induction for *clec-67*, *C14C6.5* or *K08D8.5* in the presence of *P. aeruginosa*. Taken together, these data demonstrate that RPW-24 both enhances the expression of a subset of putative immune effectors that are normally activated during *P. aeruginosa* infection and causes the induction of others that are not activated by *P. aeruginosa*. We do not, however, think that the induction of any single gene is itself responsible for the efficacy of RPW-24 since others have shown that *C. elegans* immune effectors likely function redundantly to defend against *P. aeruginosa* infection [8]. The finding that putative immune effectors are robustly induced by RPW-24 during bacterial infection provides a potential explanation why this compound provides protection against *P. aeruginosa*-mediated killing.

### Immunostimulatory Activity of RPW-24 Is Required for Its Efficacy and Is Partially Dependent on the p38 MAP Kinase Signaling Cassette

**Figure 3:**
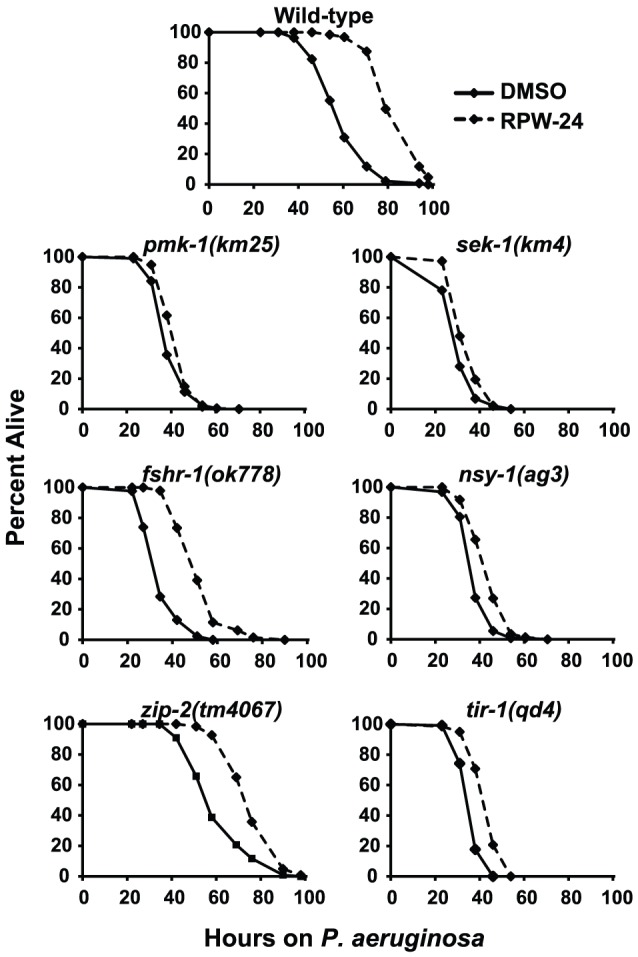
Full efficacy of RPW-24 in prolonging the lifespan of *C. elegans* infected with *P. aeruginosa* requires the p38 MAP kinase signaling cassette. *P. aeruginosa* infection assays of *C. elegans* wild-type and mutant animals exposed to 70 µM RPW-24 compared to DMSO. Data at each time point are the average of three plates per strain, each with approximately 50 animals per plate (sample sizes are given in Table S2). Data are representative of two independent experiments.

Because RPW-24 caused the induction of genes normally upregulated during bacterial infection, we reasoned that *C. elegans* immune gene activation by RPW-24 is important for its anti-infective activity and depends on conserved defense response pathways. Previous studies have identified a requirement for three *C. elegans* signaling pathways in the defense against *P. aeruginosa* infection: the p38 MAP kinase signaling cassette [10], the G-protein coupled receptor FSHR-1 [16], and the bZIP transcription factor ZIP-2 [17]. Using loss-of-function mutants, we asked whether the activity of any of these pathways is required for the efficacy of RPW-24 in treating *C. elegans* infected with *P. aeruginosa*. Indeed, we found that the magnitude of RPW-24-mediated lifespan prolongation of *pmk-1(km25)* null mutants infected with *P. aeruginosa* was significantly attenuated compared to the lifespan extension observed in compound-treated wild-type animals (Figure 3) (*P*<0.001 for the difference in the lifespan prolongation between *pmk-1(km25)* and wild-type animals in two biological replicates). In contrast to *pmk-1(km25)* mutants, *fshr-1(ok778)* loss-of-function mutants demonstrated significant lifespan extension by RPW-24 compared to *pmk-1(km25)* animals, despite the fact that *fshr-1(ok778)* animals are also hypersusceptible to *P. aeruginosa* infection (Figure 3) (*P*<0.001 for the difference in the lifespan prolongation between *pmk-1(km25)* and *fshr-1(ok778)* in two biological replicates). Likewise, the lifespan of *zip-2(tm4067)* animals infected with *P. aeruginosa* was also significantly prolonged by RPW-24, and to a greater degree than *pmk-1(km25)* (*P*<0.01 for the difference in two biological replicates). These data demonstrate that the activity of PMK-1, more so than FSHR-1 or ZIP-2, is important for RPW-24 to extend the lifespan of nematodes infected with *P. aeruginosa*. Morever, the observed reduction in the ability of RPW-24 to extend the lifespan of *pmk-1(km25)* animals is not likely to be secondary to decreased overall fitness of immunocompromised animals infected with a bacterial pathogen because RPW-24 prolongs the lifespan of *fshr-1(ok778)* animals, a strain that is also markedly hypersusceptible to *P. aeruginosa* infection (Figure 3).

The p38 MAP kinase PMK-1 functions as part of a conserved signaling cassette to regulate host innate immune responses, which involves upstream activation by the MAP kinase kinase SEK-1, the MAP kinase kinase kinase NSY-1 and the Toll-Interleukin-1 receptor TIR-1. We tested the ability of RPW-24 to extend the lifespan of nematodes with mutations in each of these genes: *sek-1(km4)*, *nsy-1(ag3)*, and *tir-1(qd4)*. As with the *pmk-1(km25)* mutants, we found that the curing activity of RPW-24 was attenuated in each of these mutant strains compared to the wild-type control (Figure 3), suggestive of a role for the entire TIR-1/NSY-1/SEK-1/PMK-1 signaling cassette in the activation of defense responses following exposure to RPW-24.

**Figure 4:**
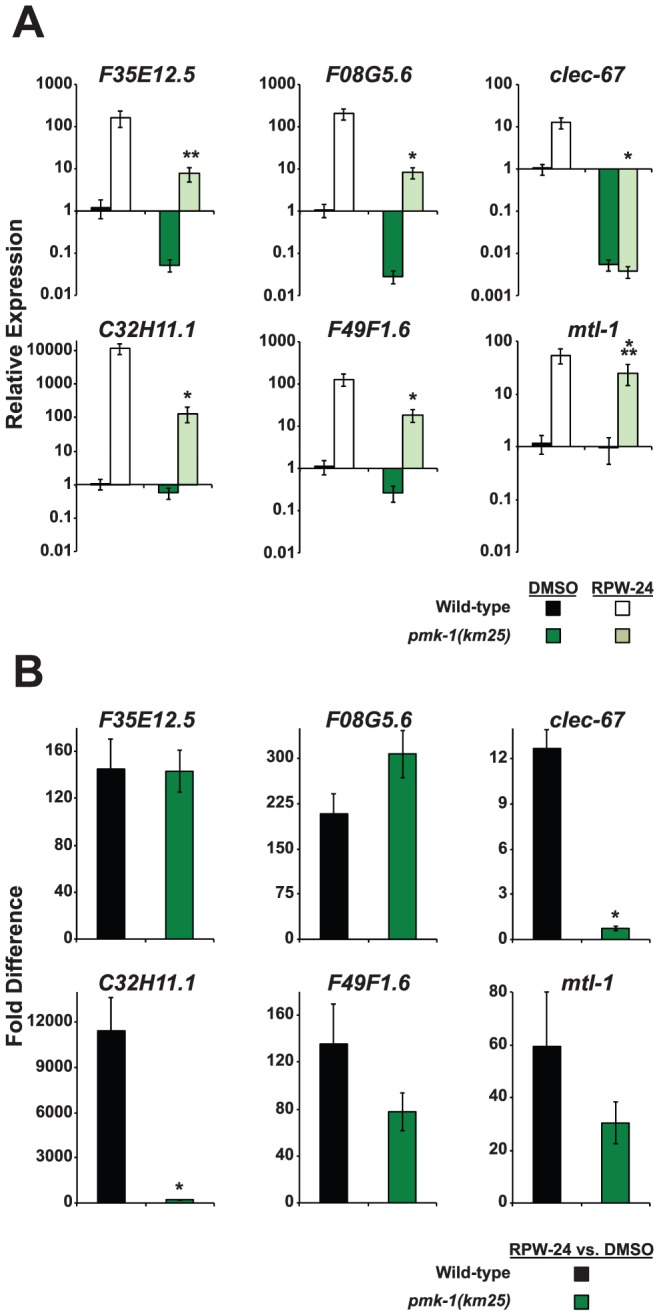
The expression levels of RPW-24–induced putative immune effectors are reduced in *pmk-1(km25)* mutants compared to wild-type animals. Six putative *C. elegans* immune effectors were analyzed by qRT-PCR in wild-type and *pmk-1(km25)* feeding on *E. coli* and exposed to either 70 µM RPW-24 or DMSO for 16 hours. Data are the average of two biological replicates each normalized to a control gene with error bars representing SEM. In (A), the data are relative to the average expression of the indicated gene in wild-type *C. elegans* exposed to DMSO. In (B), the fold change of the indicated gene above its expression in *C. elegans* exposed to DMSO is compared in wild-type and *pmk-1(km25)* mutant animals. *P<0.01, **P = 0.06, ***P = 0.09 for the comparison in (A) of relative expression levels in wild-type versus *pmk-1(km25)* animals, each exposed to RPW-24 and in (B) for the fold change in wild-type versus *pmk-1(km25)* mutant animals.

The infection assays described above show that the p38 MAP kinase pathway is required for RPW-24 to prolong the lifespan of *C. elegans* infected with *P. aeruginosa*, suggesting that RPW-24 induces the expression of immune effectors regulated by this cascade. Previous work has shown that PMK-1 coordinates the transcription of CUB-like genes, ShK toxins and C-type lectins, gene classes which are also induced by RPW-24 [8]. Indeed, we found that 14 of the 86 genes whose basal expression depends on PMK-1 [8] were induced by exposure to RPW-24, which is 14.6 fold more than expected by chance alone (*P* = 0.002)(Table S1). To test whether RPW-24 activates immune effectors in a PMK-1-dependent manner, we used qRT-PCR to determine the induction levels of six putative immune effectors in *pmk-1(km25)* null mutants exposed to DMSO and RPW-24 in the absence of pathogen. Wild-type worms were used as the control. The set of six putative immune effectors (*F35E12.5*, *F08G5.6*, *clec-67*, *C32H11.1*, *F49F1.6* and *mtl-1*) were chosen from the panel of ten genes described above on the criterion that they were robustly upregulated by RPW-24 (Figure 2A, Figure S3). Figure 4A shows that PMK-1 affects the basal expression levels of the RPW-24-induced genes *F35E12.5, F08G5.6, clec-67* and *F49F1.6* (defined as the relative expression of the gene in *pmk-1(km25)* mutants compared to its expression in wild-type animals)(Figure 4A). PMK-1 is also required for the RPW-24-mediated induction of *clec-67* and *C32H11.1* (defined as the fold difference in gene expression in the presence and absence of RPW-24 in wild-type or *pmk-1(km25)* mutant animals), and perhaps *F49F1.6* and *mtl-1*, although the differences in induction of these later two genes did not reach statistical significance (Figure 4B). Importantly, the absolute expression levels of all six of these genes following RPW-24 exposure were significantly lower in *pmk-1(km25)* animals than wild-type controls (Figure 4A). Taken together with the *P. aeruginosa* infection assays in *pmk-1(km25)* mutants (Figure 3), these data suggest that PMK-1-dependent immune effectors mediate part of the protective effect of RPW-24 in *P. aeruginosa*-infected *C. elegans*.

### RNAi Screen Reveals Role for the Transcription Factor ATF-7 in the Regulation of the RPW-24–Induced Immune Response

The phenotypic and genetic data presented above show that the p38 MAP kinase pathway is important for the RPW-24-induced modulation of *C. elegans* immune responses during *P. aeruginosa* infection. As described above, we found that 70 µM RPW-24 caused a striking increase in GFP production in the *F35E12.5::GFP* transcriptional reporter (Figure 1B). We thus reasoned that an RNAi screen could be used to find downstream regulator(s) of the RPW-24-induced immune response by identifying the genetic dependence of *F35E12.5::GFP* activation.

**Figure 5:**
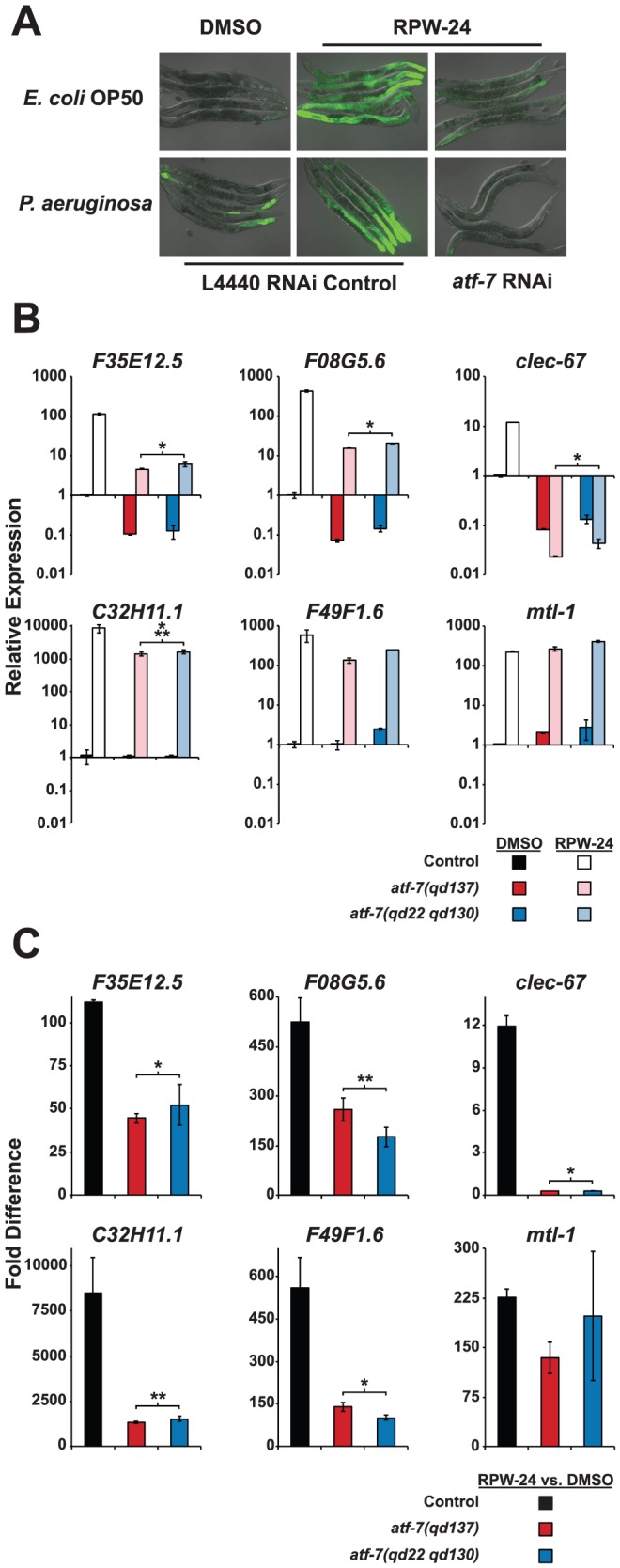
The *C. elegans* transcription factor ATF-7 regulates immune gene induction by RPW-24. (A) Fluorescence microscopy images of *C. elegans acIs101* animals, which express a *F35E12.5::GFP* transgene, exposed to the normal nematode food source *E. coli* OP50 or *P. aeruginosa* in the presence of DMSO or 70 µM RPW-24 for 16 hours at 25°C. *C. elegans acIs101* animals were raised on L4440 RNAi control bacteria or an RNAi feeder strain designed to knockdown the expression of *atf-7*. Green is GFP expression. (B and C) Six putative *C. elegans* immune effectors were analyzed by qRT-PCR in wild-type, *atf-7(qd137)* and *atf-7(qd22 qd130)* animals feeding on *E. coli* and exposed to either 70 µM RPW-24 or DMSO for 16 hours. Data are presented as the average of two biological replicates each normalized to a control gene with error bars representing SEM. In (A), the data are relative to the average expression of the indicated gene in wild-type *C. elegans* exposed to DMSO. In (B), the fold change of the indicated gene above its expression in *C. elegans* exposed to DMSO is compared in wild-type, *atf-7(qd137)* and *atf-7(qd22 qd130)* animals. An N2-derived strain carrying the *acIs219* transgene was used as the control strain because this transgene is also present in the *atf-7(lof)* strains [12]. *P<0.05, **P = 0.08, ***P = 0.09 for the comparison in (A) of the relative expression levels of both *atf-7(lof)* strains versus control animals, each exposed to RPW-24, and in (B) for the fold change in wild-type versus both *atf-7(lof)* mutants.

The basal regulation of *F35E12.5* requires PMK-1, but its induction by RPW-24 occurs in a PMK-1-independent manner (Figure 4A and 4B). We therefore anticipated that a reverse genetic RNAi screen aimed at identifying transcription factors required for the RPW-24-mediated induction of *F35E12.5::GFP* would identify genetic regulators that act either downstream of or in parallel to the PMK-1 pathway. We used a feeding RNAi library containing bacterial clones that produce double stranded RNA (dsRNA) designed to individually knockdown the expression of 393 transcription factors in *C. elegans*, corresponding to 30–50% of the transcription factors in the *C. elegans* genome [18] and screened for RNAi clones that abrogated the RPW-24-mediated induction of *F35E12.5::GFP*. Among 393 screened, we found that a single clone, corresponding to the transcription factor ATF-7, caused a striking reduction of *F35E12.5::GFP* expression when nematodes were either growing on their normal laboratory food source (*E. coli* OP50) or infected with *P. aeruginosa* (Figure 5A). ATF-7 was previously shown to function downstream of PMK-1 in the regulation of immune response genes during *P. aeruginosa* infection [12].

qRT-PCR analysis confirmed that the reduction of *F35E12.5::GFP* expression was due to the knockdown of ATF-7 and not the consequence of non-specific transgene silencing. Specifically, the absolute level *F35E12.5* expression following RPW-24 exposure was markedly reduced in two *atf-7(lof)* mutants [*atf-7(qd137)* and *atf-7(qd22 qd130)* [12]] compared to the control strain (Figure 5B), as were the levels of three other putative immune effectors (*F08G5.6*, *clec-67* and *C32H11.1*). Moreover, the basal levels of *F35E12.5*, *F08G5.6* and *clec-67* were reduced to a similar degree in the *atf-7(lof)* mutants and the *pmk-1(km25)* animals, consistent with the previously described role for PMK-1 and ATF-7 in the basal regulation of immune effectors (compare Figure 4A and Figure 5B) [12]. Interestingly, we found that ATF-7 was also required for the full induction of five of six putative immune effectors (*F35E12.5*, *F08G5.6*, *clec-67*, *C32H11.1*, and *F49F1.6*)(Figure 5C), including two genes (*F35E12.5* and *F08G5.6*) that were induced by RPW-24 independently of PMK-1 (Figure 4B).

To determine if the activity of ATF-7 is important for the efficacy of RPW-24, we tested the ability of RPW-24 to prolong the lifespan of *atf-7(lof)* mutants exposed to *P. aeruginosa*. The magnitude of lifespan extension conferred by RPW-24 was reduced in both the *atf-7(qd137)* and the *atf-7(qd22 qd130)*(Figure S4A and S4B, respectively) mutants compared to the control strain. We therefore conclude that RPW-24 stimulates the *C. elegans* immune response genes in a manner that involves both the p38 MAP kinase cassette PMK-1 and the conserved transcription factor ATF-7, consistent with the placement of ATF-7 downstream of PMK-1 [12]. Expression analysis of RPW-24-induced genes (Figure 5C) suggests that in addition to functioning downstream of PMK-1, ATF-7 receives inputs from a PMK-1 independent pathway to coordinate the induction of putative immune effectors (such as *F35E12.5* and *F08G5.6*) and the RPW-24-mediated resistance to *P. aeruginosa* infection. This conclusion is based on the finding that the RPW-24-mediated activation of *F35E12.5* and *F08G5.6* is PMK-1 independent (as opposed to their basal level of expression), but is at least partially dependent on ATF-7 (compare Figure 4B with Figure 5C). That is, the basal levels of expression of *F35E12.5* and *F08G5.6* are both PMK-1 and ATF-7 dependent, whereas the fold induction of these genes following RPW-24 is not affected in the *pmk-1(km25)* mutant, but is reduced by at least half in the *atf-7(lof)* mutants. The biological significance of this PMK-1-independent transcriptional activator is not known.

It is also interesting to note that RPW-24 exhibited an attenuated, but significant (*P*<0.001) and reproducible ability to rescue the *atf-7(lof)* mutants. Therefore, the anti-infective activity of RPW-24 may involve another immune signaling pathway, independent of both the p38 MAP kinase pathway and ATF-7. This conclusion is consistent with the observation that RPW-24 also modestly, but significantly (*P*<0.001), extends the lifespan of *pmk-1(km25), sek-1(km4)*, *nsy-1(ag3)*, and *tir-1(qd4)* mutant animals infected with *P. aeruginosa* (Figure 3), and the fact that the induction of *F35E12.5* and *F08G5.6* is not completely abrogated in the *atf-7(lof)* mutants. An alternate explanation is that RPW-24 affects virulence factor production by *P. aeruginosa* or exerts a subtle effect on growth of the pathogen.

### RPW-24 Is Toxic to *C. elegans*

In addition to inducing a preponderance of genes involved in the transcriptional response to pathogenic bacteria, RPW-24 caused the upregulation of genes involved in the detoxification of small molecules (Table S1). The microarray analysis revealed that 58 of the 269 genes activated 3 fold or more and 31 of the 57 genes activated 50 fold or more were UDP-glucuronosyltransferases (UDPs), cytochrome P450s (CYPs), glutathione-s-transferases (GSTs) or short-chain dehydrogenases (SDRs). These gene classes play integral roles in the Phase I and II detoxification of both endobiotic and xenobiotic toxins in both nematodes and mammals [19], [20]. Indeed, seven of ten CYPs, two of three UDPs, one of four GSTs, three of three carboxylesterases, and three of six C-type lectins were induced both by RPW-24 and exposure to five xenobiotic toxins [21]. Taken together, these data suggest that RPW-24 induces xenobiotic detoxification pathways in *C. elegans*.

**Figure 6:**
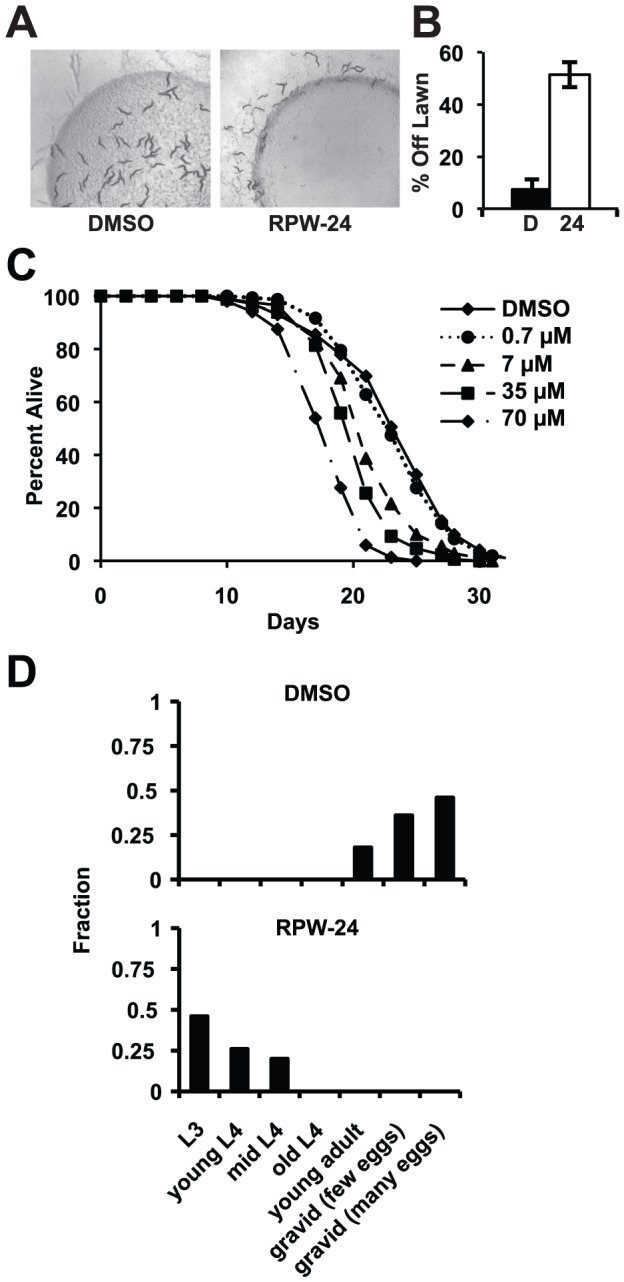
RPW-24 is toxic to *C. elegans*. (A) Wild-type *C. elegans* leave a lawn of *E. coli* containing 70 µM RPW-24 more so than DMSO after 16 hours of exposure. (B) The average percentage of wild-type *C. elegans* that were off a lawn of *E. coli* supplemented with either DMSO or 70 µM RPW-24 after 16 hours of exposure from three plates per condition [as in (A)] is presented with error bars representing SEM. *P* = 0.002 for the comparison of the two conditions. (C) RPW-24 shortens the lifespan of wild-type *C. elegans* in a dose-dependent manner. Data at each time point are the average of three plates per strain, each with approximately 50 animals per plate (sample sizes are given in Table S2). (D) RPW-24 slows development of wild-type *C. elegans*. Developmental stage was accessed in 50 animals per treatment group and presented as the percentage of the population that was at the indicated development stage after 65 hours of incubation at 20°C.

To determine if RPW-24 adversely affects wild-type nematodes growing in the absence of pathogen, we first used a behavioral assay designed to study the aversion response of *C. elegans* to xenobiotic toxins [22]. The addition of some poisons to the center of small lawns of non-pathogenic *E. coli* causes *C. elegans* animals to leave the lawn, presumably to minimize toxin exposure. Interestingly, we observed a significant aversion response to 70 µM RPW-24 (Figure 6A and 6B). After 16 hours, 51% of the nematodes had left the lawn containing RPW-24, whereas only 7% of animals left a control lawn (*P* = 0.002; Figure 6B). Next, we conducted a lifespan assay on nematode growth media supplemented with either DMSO or varying concentrations of RPW-24 and found that RPW-24 shortened *C. elegans* lifespan in a dose-dependent manner (Figure 6C), with 70 µM RPW-24 resulting in a 24% reduction in median lifespan. Interestingly, we observed lifespan shortening only at compound concentrations that rescued *C. elegans* from *P. aeruginosa* infection [7, 35 and 70 µM, but not 0.7 µM]. We also found that 70 µM RPW-24 slowed the development of animals when they were exposed at the first larval stage (L1)(Figure 6D).

In *C. elegans*, oxidative stress is a potent inducer of phase II detoxification genes. Moreover, the p38 MAP kinase PMK-1 regulates the cellular response to oxidative and arsenite stress, but through the transcription factor SKN-1, not ATF-7 [12], [23]–[25]. We therefore wondered whether RPW-24 might confer protection against arsenite stress. We found, however, that 70 µM RPW-24 did not protect nematodes exposed to 5 mM sodium arsenite for 16 hours at 20°C (Figure S5). In fact, the toxicity of RPW-24 and sodium arsenite were synergistic in this assay, resulting in nearly 100% mortality of wild-type nematodes exposed to both compounds (Figure S5).

While RPW-24 confers a survival advantage for nematodes infected with pathogenic bacteria, these data demonstrate that it is toxic to *C. elegans* growing under standard laboratory conditions. Whether this toxicity is a consequence of direct effects of the compound on *C. elegans* or hyper-activation of the immune system by RPW-24 is unknown.

## Discussion

We hypothesized that a *C. elegans*-based screen for novel anti-infectives would identify small molecules that act by stimulating the host immune response. Four lines of evidence allow us to conclude that RPW-24 protects *C. elegans* from bacterial infection by inducing the production of putative immune effectors via an evolutionarily conserved immune pathway. First, RPW-24 confers a survival advantage for nematodes infected with the Gram-positive bacteria *E. faecalis* and the Gram-negative bacteria *P. aeruginosa* at doses that do not inhibit *in vitro* growth of these pathogens. Second, whole genome microarray analysis demonstrates that RPW-24 induces a transcriptional response in *C. elegans* in the absence of pathogen exposure. We observed that 26% of the genes induced 3-fold or greater are also induced by infection with *P. aeruginosa*. Moreover, we showed that the RPW-24-mediated induction of several putative immune effectors is enhanced in the context of *P. aeruginosa* infection. Third, using *C. elegans* animals deficient in immune pathway signaling in pathogenesis assays and gene expression analyses, we demonstrated that the TIR-1/NSY-1/SEK-1/PMK-1 cascade in *C. elegans* is required for RPW-24 to exert its full effect. Lastly, an RNAi screen indicated that the transcription factor ATF-7 controls the RPW-24-mediated induction of a putative immune effector gene. The ability of RPW-24 to promote survival of *P. aeruginosa*-infected nematodes is partially dependent on this transcription factor. ATF-7 has been shown to function downstream of the MAPK PMK-1 to activate the expression of immune effectors [12]. Here we show that ATF-7 also activates the expression of genes independently of the PMK-1 p38 MAPK pathway. In summary, these data strongly suggest that immune gene activation by RPW-24 is required for its protective effects. While it is theoretically possible that RPW-24 confers protection for *C. elegans* by also inhibiting virulence factor production in both *E. faecalis* and *P. aeruginosa*, we feel this is less likely given that clear effects of RPW-24 on the nematode and the evolutionary diversity between these bacterial pathogens.

An unexpected observation in our transcriptome profiling analysis was that RPW-24 caused the dramatic induction of genes involved in the detoxification of small molecules, including UDP-glucuronosyltransferases (UGTs), cytochrome P450s (CYPs), glutathione-s-transferases (GSTs) and short-chain dehydrogenases (SDRs). In mammals, these gene classes act together to detoxify xenobiotic small molecules via two successive reactions [19]. Phase I reactions involve the addition of chemically reactive functional groups to the toxins and are predominantly mediated by CYPs and SDRs. In Phase II, UGTs and GSTs add side groups that increase the solubility of small toxic molecules, which aides in their excretion. These mediators of detoxification are highly conserved throughout evolution and are present in *C. elegans* [20], [26], [27]. Their marked induction by RPW-24 suggests that this compound may be recognized as a toxin. Consistent with this hypothesis, RPW-24 caused a strong behavioral avoidance phenotype, shortened nematode lifespan, and delayed the development of nematodes growing on non-pathogenic bacteria.

We have identified a low molecular weight molecule that can potently activate the innate immune response of *C. elegans*. The target of this compound is not known, nor are the mechanisms that act upstream of the p38 MAP kinase cassette to trigger the RPW-24-mediated immune activation in *C. elegans*. Indeed, it also unclear how any of the *C. elegans* immune pathways, including the p38 MAP kinase cassette, are activated during bacterial infection. Recently, several investigators have shown that the nematode monitors the integrity of cellular processes as a means to detect pathogen invasion, and to trigger defense responses and behavioral avoidance phenotypes. McEwan et al. [28] and Dunbar et al. [29] each found that the inhibition of translation by a bacterial toxin induced a protective immune response in *C. elegans* that was dependent on the p38 MAP kinase and ZIP-2 pathways. Similarly, Melo et al. showed that disruption of core cellular processes, such as translation, mitochondrial respiration and proteasome function, by bacterial toxins induced a behavioral avoidance phenotype [22]. Thus, it is possible that the toxic effects of RPW-24 trigger immune response pathways and a behavioral aversion response in an analogous manner. Alternatively, RPW-24 itself could directly activate immune pathways in *C. elegans*.

In this study, we have demonstrated the utility of using small molecules in conjunction with classical epistasis analysis and RNAi screens to dissect immune signaling pathways in *C. elegans*. We therefore hypothesize that RPW-24 can be used as a tool in additional genetic analyses both to identify the target(s) of this small molecule and to determine mechanism by which the p38 MAP kinase pathway is activated by RPW-24, which may offer insights into how *C. elegans* detects bacterial pathogens.

The World Health Organization has declared that antimicrobial-resistant pathogens are one of the three greatest threats to human health. Exacerbating this problem is the striking absence of novel antibiotics in the development pipeline. In 2008, there were only 16 antimicrobial compounds in late stage clinical trials and only one of these agents had a novel mechanism of action [30]. Furthermore, all of these agents target some aspect of bacterial replication or metabolism. Indeed, it has been suggested that the obvious bacterial targets amenable for antimicrobial drug design have been exhausted [31]. Identifying host-acting small molecules that modulate innate immune responses is a promising approach to identify novel antimicrobial therapies [32]. In theory, such agents should place minimal selection pressure on bacteria to acquire resistance determinants and could have broad-spectrum antimicrobial activity. Agonists of the mammalian Toll-like and NOD-like receptors are among the most promising compounds that are being explored for this purpose [32], [33]. We propose that *C. elegans*-based compound screening assays can be used to mine large chemical libraries for additional small molecule anti-infectives that act by stimulating host immune defenses. Whether or not these host-acting small molecules will be effective for the treatment of bacterial infections in mammals, genetic dissection of the *C. elegans* signaling pathways activated by such compounds may suggest strategies for the development of new classes of antimicrobial therapies.

## Materials and Methods

### *C. elegans* Strains

*C. elegans* were maintained and propagated on *E. coli* OP50 as described [34]. The *C. elegans* strains used in this study were: N2 Bristol [34], *pmk-1(km25)* [10], *sek-1(km4)* [10], *nsy-1(ag3)* [10], *tir-1(qd4)* [11], *atf-7(qd137)* [12], *atf-7(qd22 qd130)* [12], *fshr-1(ok778)* [16], *zip-2(tm4067)* [17] *glp-4(bn-2)* [35], AU78 [*agIs219 (pT24B8.5::GFP::unc-54*-3′UTR p*ttx-3::GFP::unc-54*-3′utr)] [11] and AY101 [*acIs101*[*pDB09.1(pF35E12.5::GFP*); pRF4(*rol-6(su1006)*)] [13].

### *C. elegans* Bacterial Infection and Other Assays

Slow-killing *P. aeruginosa* solid media infection assays were performed as previously described [15] with some modifications. A single colony of *P. aeruginosa* PA14 was innoculated into 3-mL of Luria-Bertani (LB) media and allowed to incubate at 37°C for 14 to 15 hours. 10 µL of this culture was added to 35-mm tissue culture plates containing 4 mL of slow kill agar supplemented with 1% DMSO and the indicated conentration of RPW-24. Others have shown that this concentration of DMSO has little effect on growth or development of *C. elegans* [36], [37]. Plates were incubated for 24 hours at 37°C and 24 hours at 25°C. *C. elegans* lifespan assays were conducted on nematode growth media (NGM) supplemented with the indicated concentraion of RPW-24 and seeded with OP50. The sensitivity of RPW-24-treated wild-type animals to oxidative stress was determined using 5 mM sodium arsenite following a previously described protocol [12]. For the infection, lifespan and sodium arsenite assays, 0.1 mg/mL 5-fluorodeoxyuridine (FUDR) was added to the media 1 to 2 hours before the start of the assay to prevent progeny from hatching. Approximately 50 L4 staged nematodes were picked to each of three or four assay plates per experimental condition. Animals were scored as live or dead on a daily basis by gently touching them with a platinum wire. Worms that crawled onto the wall of the tissue culture plate were eliminated from the analysis. The *P. aeruginosa* killing assays were conducted at 25°C. The lifespan and sodium arsenite assays were performed at 20°C. The sample sizes for each of these experiments are given in Table S2. For the experiments with the *atf-7(lof)* mutants, we used an N2-derived strain carrying the *acIs219* transgene (AU78) as the control strain because this transgene is also present in the *atf-7* mutant strains [12]. The *C. elegans* liquid media infection assay used to screen the 31 compounds for those with activity against *P. aeruginosa*-infected nematodes was developed in our laboratory and was conducted in either 384 or 96 well plates using *glp-4(bn-2)* animals (Kirienko, NV and Ausubel FM, unpublished data). Growth of *P. aeruginosa* in the presence of RPW-24 was determined by inoculating 1.0×10^4^ bacteria in liquid slow-kill media containing either 70 µM RPW-24 or DMSO and allowing the culture to grow at 37°C in a roller drum. At the indicated time points, 10 µL of the culture was removed and CFUs were determined by plating serial dilutions.

The propensity of wild-type *C. elegans* to leave a lawn of bacteria supplemented with RPW-24 was assayed using a previously described protocol [22]. Briefly, 6-well tissue culture plates containing NGM were seeded with concentrated *E. coli* OP50. 70 µM RPW-24 or an equal volume of DMSO was added to the center of the *E. coli* lawn and allowed to dry. 70 to 90 young L4 animals were added to the center of the lawns and animals were scored as either on or off the lawn after 16 hours incubation at room temperature. To determine if RPW-24 slowed the development of wild-type animals, 70 µM RPW-24 or DMSO was added to NGM plates and seeded with OP50. L1 staged animals, synchronized by hypochlorite treatment, were added to these plates and allowed to incubate at 20°C for 65 hours. Developmental stages of 50 animals per treatment group were determined by microscopic examination of the gonad.

### *C. elegans* Microarray Analysis

N2 animals were synchronized by hypochlorite treatment. Arrested L1s were plated on 10 cm NGM plates seeded with *E. coli* OP50 and grown at 20°C until the late L4 larval stage. Animals were incubated for 15 hours at 15°C in 2 mL of liquid S-basal complete medium [38] containing 70 µM RPW-24 or DMSO and supplemented with *E. coli* OP50. The final concentration of DMSO in both samples was 1%. RNA was extracted from three biological replicates using TRI Reagent (Molecular Research Center) according to the manufacturer's instructions and purified using an RNeasy column (Qiagen). RNA samples were prepared and hybridized to Affymetrix full-genome GeneChips for *C. elegans* at the Harvard Medical School Biopolymer Facility (Boston, MA) following previously described protocols [5], [8] and instructions from Affymetrix. Data were analyzed using GenePattern version 2.0 software using GC-RMA and quantile normalization [39]. Conditions were compared using GenePattern to determine the fold change between conditions for each probe set and to generate a *P* value using a modified *t*-test. Probe sets were considered differentially expressed if the fold change was 3-fold or greater (*P*<0.025).

### Quantitative RT–PCR (qRT–PCR) Analyses

Animals of the indicated genotype were treated and RNA was extracted as described for the microarray analysis. For the experiments with the *atf-7(lof)* mutants, we used strain AU78 as as the control strain [12]. For gene expression analysis of nematodes on solid media, 70 µM RPW-24 or DMSO was added to 20 mL nematode growth media in 10 cm petri dishes seeded with *E. coli* OP50. For qRT-PCR studies of nematodes infected with *P. aeruginosa*, 20 mL of slow killing media was added to 10 cm petri dishes containing either DMSO or 70 µM RPW-24. Plates were seeded with either 250 µL of *E. coli* OP50 or 50 µL *P. aeruginosa* diluted in 200 µL LB, each from overnight cultures. The plates were incubated for 24 hours at 37°C and 24 hours at 25°C. Old L4/young adult animals were added to the assay plates and incubated at 25°C for eight hours. RNA was reverse transcribed to cDNA using the Retroscript kit (Ambion). cDNA was analyzed by qRT-PCR using a CFX1000 machine (Bio-Rad) and previously published primers [8]. All values were normalized against the control gene *snb-1*, which has been used previously in qRT-PCR studies of *C. elegans* innate immunity [5], [8], [9], [12], [40]. Analysis of the microarray expression data revealed that the expression of *snb-1* did not vary under the conditions tested in the experiment. Fold change was calculated using the Pfaffl method [41].

### Feeding RNAi Screen

The RNAi screen of 393 transcription factors was conducted using RNAi clones from the Ahringer and Vidal RNAi libraries and an established protocol [42]. The RNAi clone for *atf-7* was developed by Shivers et al [12]. Briefly, overnight cultures of feeding RNAi clones were added to each well of a 96-well RNAi plate and allowed to grow at room temperature overnight. 40–60 L1 staged *acIs101* animals, which express a *F35E12.5::GFP* transgene, were added to each well and allowed to grow for two days at 20°C until they were at the L4 or young adult stage. Animals were then washed from the RNAi plates into S-basal complete media [38] containing 70 µM RPW-24. All experiments with feeding RNAi used a *gfp* RNAi as the negative control, which resulted in no visible GFP expression in *acIs101* transgenic animals. *acIs101* animals treated with the empty vector L4440 and exposed to 70 µM RPW-24 was used as the positive control. Animals were scored for GFP expression following photograph of each well using an Image Xpress Micro microscope (Molecular Devices Corporation, Sunnyvale, CA).

### Microscopy

Nematodes were mounted onto agar pads, paralyzed with 10 mM levamisole (Sigma) and photographed using a Zeiss AXIO Imager Z1 microscope with a Zeiss AxioCam HRm camera and Axiovision 4.6 (Zeiss) software.

### Statistical Analyses

Differences in survival of *C. elegans* animals infected with *P. aeruginosa* on slow-killing assay plates were determined with the log-rank test. To determine if the increase in survival conferred by RPW-24 treatment was different in one population compared to another [for example, in wild-type versus *pmk-1(km25)* animals], we examined the difference in the effect of RPW-24 treatment on the hazard in each group using a Cox proportional hazard model (Stata11, Stata, College Station, TX). Fold changes in the qRT-PCR analyses and the differences in survival in the arsenite assays were compared using unpaired, two-tailed student *t*-tests. When comparing microarray datasets, the overlap expected by chance alone was determined in 50 groups of randomly selected *C. elegans* genes using Regulatory Sequence Analysis Tools (http://rsat.ulb.ac.be/rsat/), a technique that has been used for similar analyses [43]. *P* values were determined using chi-square tests.

### Accession Numbers

Accession numbers for the genes and gene products mentioned in this paper are given for Wormbase, a publicly available database that can be accessed at http://www.wormbase.org. These accession numbers are *pmk-1 (B0218.3), nsy-1 (F59A6.1), sek-1 (R03G5.2), atf-7 (C07G2.2), zip-2 (K02F3.4), fshr-1 (C50H2.1), skn-1 (T19E7.2), C32H11.1, F35E12.5, F08G5.6, C29F3.7, K08D8.5, F49F1.6, C14C6.5*, *lys-7 (C02A12.4)*, *clec-67* (*F56D6.2*) and *mtl-1 (K11G9.6)*. The microarray dataset can be downloaded from the National Center for Biotechnology Gene Expression Omnibus (GEO; http://www.ncbi.nlm.nih.gov/geo). The accession number for these data is GSE37266.

## Supporting Information

Figure S1 The compound structures of the anti-infective small molecules presented in Table 1.
(EPS)

Figure S2 RPW-24 reduces the burden of *P. aeruginosa* in the *C. elegans* intestine. Wild-type nematodes exposed to either DMSO or 70 µM RPW-24 were photographed 40 hours after infection with *P. aeruginosa*. At 40 hours after infection, greater than 95% of animals are still alive in both treatment groups (Figure 1C). Images from the proximal (top) and mid (bottom) portions of the intestine are presented. Arrowheads outline the intestinal lumen and the arrow points to the pharyngeal grinder. Individual *P. aeruginosa* can be seen within the *C. elegans* intestine in the picture on the top left. The scale bar equals 10 µm.
(EPS)

Figure S3 RPW-24 induces the transcription of putative immune effectors. 10 putative immune effectors that were induced in the microarray analysis (Figure 2A) were studied by qRT-PCR in RPW-24 and DMSO-exposed animals on solid and in liquid media. The fold change of the indicated genes in animals exposed to RPW-24 versus expression in DMSO-exposed animals in the transcriptome profiling analysis is indicated with gray bars. qRT-PCR data are plotted versus expression of the indicated genes in *C. elegans* exposed to DMSO on the indicated media and are presented as the average of two biological replicates each normalized to a control gene with error bars representing SEM.
(EPS)

Figure S4 The full efficacy of RPW-24 requires the *C. elegans* transcription factor ATF-7. *P. aeruginosa* infection assay of wild-type *C. elegans* compared to *atf-7(qd137)* (A) and *atf-7(qd22 qd130)* mutant animals (B) exposed to 70 µM RPW-24 and DMSO. Data at each time point are the average of three plates per strain, each with approximately 50 animals per plate (sample sizes are given in Table S2). The lifespan extension conferred by RPW-24 in wild-type animals was significantly greater than that observed in *atf-7(qd137)* and *atf-7(qd22 qd130)* animals (*P*<0.001 for both comparisons). Data are representative of two independent experiments. An N2-derived strain carrying the *acIs219* transgene was used as the control strain because this transgene is also present in the *atf-7(qd137)* mutant [12].
(EPS)

Figure S5 RPW-24 does not cause resistance to arsenite. Animals were treated with 5 mM sodium arsenite and 70 µM RPW-24 (24) or DMSO (D) for 18 hours. Shown is the average survival of animals on three plates per condition, each with approximately 50 animals per plate (sample sizes are given in Table S2). The error bars are SEM. *P*<0.01 for the comparison in survival.
(EPS)

Table S1 Differentially expressed genes following RPW-24 treatment identified in a genome-wide transcriptional profiling experiment. Presented are the lists of Affymetrix probe sets, the corresponding gene names, the fold change and the *P* value for all probe sets whose expression changed more than three-fold (*P*<0.025) in wild-type nematodes exposed to RPW-24 versus DMSO. A summary of the data is presented at the bottom of the worksheet.
(XLSX)

Table S2 Sample sizes for the infection assays, the lifespan assay and the arsenite assay. Sample sizes do not include censored animals.
(XLSX)
